# The retention of physicians to primary health care in Brazil: motivation and limitations from a qualitative perspective

**DOI:** 10.1186/s12913-018-3813-3

**Published:** 2019-01-22

**Authors:** Lyane Ramalho Cortez, Eliana Costa Guerra, Natércia Janine Dantas da Silveira, Luiz Roberto Augusto Noro

**Affiliations:** 1 Federal University of Rio Grande do Norte - UFRN, Post graduate collective health program, Av. Sen. Salgado Filho 3000, Natal, 59064-741 RN Brazil; 2 Federal University of Rio Grande do Norte - UFRN, Departament of Group Health - UFRN, Av. Sen. Salgado Filho 3000, Natal, 59078-970 RN Brazil

**Keywords:** Evaluation of health programs and health projects, Primary health care, Unified health system, Medical education

## Abstract

**Background:**

This study analyzed the reasons why physicians migrated from the Program to Value Primary Healthcare Professionals (PROVAB) to the *Mais Médicos* (More doctors) Program in 2015, opting to become physicians of the Primary Health Care.

**Methods:**

The focal group techniques were used consisting of six doctors that made the option to remain in the PHC, being the results analyzed through content monitoring as well as the respective categories, which were identified as theme analysis.

**Results:**

It was evidenced that the physician’s retention on primary care has a strong relationship with the acquisition of knowledge that is consistent with the context and the health needs of the population. Thus, appropriate competencies for the management of the different biopsychosocial characteristics of the territories, in consonance with abilities that use the communication centered on the patient as well as on the interdisciplinarity, producing health projects that include the uniqueness of the people, essential factor for the daily confrontation of the work of these physicians, strengthened by the training component of the program. Personal factors related to the empathetic profile with this level of care and the possibility of continuous improvement, besides the factors related to the adequate infrastructure and organizational climate with guaranteed salary in keeping with the complexity of Primary Care, positively influenced the permanence of these professionals and were drivers of changes in healthcare and management in the health units they were related to. The bond created with the team and patients was a great satisfaction factor for the professional, being identified by them as a possibility to make a difference in the treatment process of patients, strengthening the promotion of a healthier life through health education.

**Conclusions:**

PROVAB had an unparalleled contribution to the qualification of care in Primary Health Care, contributing to the birth of a new logic of medical training in Brazil. Its effective cooperation with the consolidation of the Mais Médicos Program, still in progress, is a great movement of resistance to the disarticulation and deconstruction of Unified Health System (SUS) which is in charge of public health in Brazil.

## Background

There is a historical difficulty in providing and setting up doctors in several countries around the world. Approximately half the population of the world live in rural areas, though this population is only attended by a quarter of the total doctors workforce, being estimated that 1 billion of people doesn’t have access to the essencial health care. In South Africa, 46% of the population live in rural areas, but only 12% of the doctors work in these territories [1].

This problem is mainly caused by the chronic and growing deficit of doctors due to the increase in the population’s health needs and the insufficient number of doctors trained in the creation of new work centers. Countries such as the United States and the United Kingdom maintain rates of foreign doctors working in their country around 25 and 27%, respectively, meaning that even developed and rich countries may have deficits of this professional in rural and less privileged areas. Also, 50% of the world’s doctors are located in areas with less than a fifth of the population and the regions of extreme vulnerability are still in need of this professional [1–3]. Monetary incentives, debt relief contracted by doctors in undergraduate courses, for providing service after graduating in difficult areas are some of the strategies that these countries use as a way of solving this challenge [4, 5].

In Brazil, the overview is not different, and several policies have already been implemented as a solution to this problem [6, 7]. From 2011, this challenge starts to be seen as a social issue by the government and has been the object of several public policies that were understood to be directly related to the doctor’s retention on Primary Health Care (PHC). National debates guided the theme of attraction, provision and retention of medical professionals in Brazil’s PHC in order to understand the main factors related to this difficulty, such as: [8] the fragile capacity of contracting these professionals by the Brazilian municipalities in the face of high fiscal instability of budgets, culminating in precarious contractual relationships; the territorial concentration of medical schools in the capitals and developed towns of the country and the training with emphasis on medical specialties with insufficient insertion in the basic care network of the Unified Health System (SUS); and the poor working and living conditions offered to doctors and their families, with professional isolation and lower chances of specialization and career advancement in these remote municipalities. [8, 9].

Thus, in September 2011, the Ministry of Education and the Ministry of Health (MS) launched the Interministerial Ordinance 2087 / 2011, which established the Program to Value Primary Healthcare Professionals (PROVAB) [10, 11]. Besides to the provision of medical professionals in the territory during a one-year period, PROVAB advocated a training component with face-to-face and distance teaching supervision developed by Higher Education Institutions (HEI), as well as requiring a Specialization Course in Family Health during the period in which the doctor was a student of the program, so this professional could be better able to serve the population of the PHC, besides being under a network of face-to-face and distance support for the main difficulties of daily dealing. Considering the doctor’s intention to submit to the medical residency selection process, the professional was evaluated in a procedural manner and should perform satisfactorily to achieve the bonus in the final grade of this selection process. Accordingly, the bonus was also thought as a way to attract the university graduate to the PHC [10, 11].

It was expected that PROVAB would contribute to a continuity of learning, after completing the course of Medicine in the teaching-service-community integration [12], helping with the qualification of a generalist, humanist professional with a critical and reflexive stance, able to work as a team, having the SUS as a great formative scenario [13, 14], a profile that is recommended by the national curricular guidelines of the medical course [13, 14], which reinforces the strategic importance of PROVAB and its effective contribution to the formation of a professional who is more focused on the health needs of the population [11, 12].

PROVAB stands out because it is a strategy which provides the unity of Work Management and Health Education, whose presupposition was to invest in a set of measures that sought to qualify and value the work performed by the primary health care teams, offering both physical and financial working conditions and access to in-service training [12, 15]. Thus, in 2013, the *Mais Médicos* (More doctors) Program (PMM), regulated by Law 12,871 [16], emerges as a response to this issue, taken as a social problem, much of which is still due to society’s growing demand for improving Health Care services as well as the presence of doctors whre they are needed. [2]. The PMM was structured in three action axes that had as a presupposition, working together, to increase the supply of doctors and to improve the assistance conditions in the Brazilian municipalities [9].

PROVAB’s surprise effect came in 2015 when doctors began to consider their stay for more than a year in the PHC, in the territories where they were already working as doctors of a Family Health Team. This time, the MS allowed the doctors to stay in the territories where they were already linked, as a means of maintaining these professionals at the PHC. This migration that was titled as precedence for the PMM was progressively adopted by PROVAB doctors, so the number of those who decided to extend the time spent acting through Federal Government incentives more than quadrupled between 2014 and 2016, reaching 55% of the total of graduates in PROVAB in 2016 [15].

The goal of this research was to analyze the factors and motivations that led doctors to move from PROVAB to PMM in the year 2015, choosing to continue as PHC doctors. There are studies which show the difficulty to lead the doctors to work permanently at the PHC [3, 15]. Therefore, the literature that searches for understanding the reasons of the retention at PHC, still needs deepening, with a view to contribute to the solution of these problems in Brazil, being an example to other countries in similar situation, since WHO recommends that, in order to face this problem, it’s essential to know deeply the reality of the Health workers [3]. To that end, it’s necessary to analyze the factors which affect the health staff decisions, once they move to rural or remote places, but also to remain or leave these areas. Without the knowledge the choices and interventions are not sustainable and, in general, they are rejected by health professionals [5, 17, 18].

## Methods

The present study is a qualitative research that aimed to understand the phenomenon that led PROVAB physicians to remain in PHC.

The focal group technique was chosen for data collection because it is characterized as a group dynamics technique involving subjects with similar characteristics in relation to the object of the research, aiming to foment discussions that allow an exchange of knowledge and experiences among participants [19]. Focal group favors interaction, allows the emergence of social representations in their contents and constituent movements [20], an essential tool to understand the involvement of research subjects with the advances provided by the PROVAB [19, 20]. The study was developed with physicians who participated in the PROVAB in the municipality of Natal in the year 2015 and who chose to continue their activities within the PMDB.

The preliminary approach of doctors was carried out by an invitation letter to 18 doctors who took precedence in 2015 of PROVAB for PMM, whose contents contained the basic explanations and the purpose of the research, and 6 of them were selected to participate in the research. From the total, six accepted to take part in the research, six didn’t reply to the invitation letter and six didn’t show interest in participating, for personal reasons. The focal group took part in a specially prepared room for the development of the collective interview at the Federal University of Rio Grande do Norte, located in a central region of the municipality of Natal, aiming to permit the participation with confidentiality and without external factors interference. This way an intentional sampling was chosen for the fact that it permits the choice of key information due to the positions of the doctors in relation to the study focus [21]. The team that carried out the process was composed of a moderator, an observer and a rapporteur, who welcomed the participants and conducted an initial introduction of each person in the meeting. The process was explained emphasizing mutual trust to be shared by all as well as the importance and significance of all contributions, including those that are divergent; the perspective that the opinions were impressions and that there are no right or wrong attitudes; the duration of the activity; the guarantee of confidentiality and finally the need to remain in the room throughout the process. In addition to recording the speeches, the rapporteur made a written record of the discussion, leaving the observer responsible for identifying symbolic, affective and attitudinal elements present during the focal group meeting. The process was supported by a semi-structured interview script that addressed the importance of the PROVAB for the permanence of the physicians in PHC, undergraduate training on PHC, the role of service infrastructure and the factors that influenced the participants to remain in the PHC. Besides the focal group meeting, a semi-structured interview was conducted to characterize the participants.

The content analysis technique was used for qualitative analysis of the data. This technique seeks to understand the meanings in an objective, scientific and systematic way, giving the researcher an in-depth interpretation on the theme and coherent with the studied reality [22], based on the transcribed lines. In connection with content analysis, the thematic modality was used. This was composed of three stages: a) Pre-analysis, including the elaboration of fundamental indicators for the understanding of the investigated material through a floating reading, the creation of the corpus and the formulation and reformulation of hypotheses and objectives; b) Material exploration, in which similarities and differences were identified, categorized and organized in order to lead to an understanding of the text, and c) Processing of the obtained results and their interpretation [22]. The categories of thematic analysis were identified through the reading of the transcribed material, according to the objectives of the study [22]. In order to present the results, and in order to conceal the identity of the participants, it was agreed to identify them according to the names chosen by the own physicians during the development of the focal group meeting, based on an adjective that represented the professional. Before the development of the focal groups, a consent form was obtained from the institution to which the doctor was linked and where he/she was developing the PROVAB. The analysis and interpretation of the contents were conducted by three of the article’s authors, in a way that the final result was extensively constructed and revised by all the authors involved in the research.

The study complied with Resolution n^o^ 466 of December 12, 2012, of the National Health Council, and was guided mainly by ethical principles, ensuring confidentiality and protection of participants, and was approved through the Opinion 1,599,703 of the Research Ethics Committee of the Onofre Lopes Hospital, in June 17, 2016. For participating in the research, all physicians signed an Informed Consent Form as a way to preserve the confidentiality and guarantee of the use of the data obtained exclusively for the purposes of the research.

## Results

Among the participant doctors of the focal group, all of them had been graduated less than 5 years before and were from 27 to 33 years old, consisting of one male and five female. Two from the six doctors were married by the time of the research. All the doctors came from urban areas. The six Health units where the doctors work are located in extreme poverty and social vulnerability areas in Natal. Half the doctors, as they enter the program, intend to do a selection in Medical Residency after a year, using the Program bonus in different specialties and the other half is still unsure about which direction to follow in life.

The group was emphatic when affirming that their practice in PHC during graduation was insufficient and they were still unprepared to join a Family Health Strategy (FHS) team, a strategy considered a priority because it contributes to the expansion, qualification and consolidation of the PHC in Brazil, increasing the resolution and impacting the health situation of people and communities, besides providing an important cost-effectiveness ratio [10, 17].

After reading the transcripts, four categories were identified and characterized as a) The doctor’s training - before and after PROVAB; b) Personal factors in physicians’ retention in PHC; c) Working factors in doctors’ retention in PHC; d) The bond as a singular component of the retention;

### The doctor’s training - before and after PROVAB

The results showed that the training of these professionals to work in PHC was heterogeneous and depended on the institution in which the professional was trained. Thus, the participants’ training varied considerably. The doctors attended the graduation in different HEIs, mostly public. In some of them, the training did not include PHC -related competencies throughout the course.

*“My training was practically within a health center from the first period until the last one. I learned a lot about politics, about funding, more than, for example, about psychiatry. We leave without knowing anything about the clinic ” (Doctor 1)*

*“My college is a pioneer in the collective health field. That project and I do not know if you've heard of it, recovering the communities' springs, then, it's a rural boarding school. We move to the city in the 11* ^*th*^ *period... and we work as a family doctor and our Undergraduate Final Project is like a portfolio of everything we did there and it worked perfectly well. The nurse knew every person around the corner. ” (Doctor 2)*

When teaching has an emphasis on PHC, it is a closer approach to the Unified Health System (SUS), public services, and the population’s real health needs, which is a determining factor for the future professional practice of this student, as it can be evidenced by transcribed speech:

*“I graduated from the University [XXXX] and there I only had a period in Primary Care, although there are four Public Health subjects. But, my training was in a hospital.” (Doctor 3)*

*“In my college, I was not taught how to deal with the team, with all categories, how to work from equal to equal.” (Doctor 4)*

The entry of these doctors into the world of work, experiencing PHC during PROVAB and later in the PMM contributed to the acquisition of skills that had not been acquired during graduation. Cross-disciplinary and interdisciplinary relationship with other professionals, the learning of concepts related to work management in the PHC and the planning of actions according to the demands of the territory were competencies contemplated in this training process. This demonstrates how the real and relational insertions of experience, are products that must be pursued in the training of the doctor. The testimonies are revealing in this sense:

*“I think he added a lot of teamwork here. In college, you do not teach how you deal with the team, it does not teach you how to deal with all categories, how to work from equal to equal at least in the institution that I graduated, it is very doctor-centered.” (Doctor 4)*

*“PROVAB added practically 100% of knowledge in terms of planning, management for Basic Care and the functioning of a team.” (Doctor 5)*

*“... We learned a lot, health at school... they go through all the programs,health assistance for the child, the woman and the elderly, even if you did not do the conclusion work on that theme, you had to know all. However, sometimes they exaggerated without understanding that here the reality was different.” (Doctor 3)*

### Personal factors of physician retention in PHC

The research shows that to act as a PHC doctor, besides to monetary stimulus and extra punctuation in the selection process for the Residence, such as PROVAB, a certain profile must be imperative, consistent with the problems faced by a FHS team. Thus, the bonus provided by PROVAB was understood as a stimulus for the doctor’s entry into the Program, but in order to work as a factor of maintaining this professional in the PHC, it must be in line with the professional profile to work at that level of attention. This understanding is evidenced in the speech of the research physicians:

*“If you entered the program with the 10% thought [subsidy for the Medical Residency] giving importance to having a good salary and you do not have the profile of family doctor you do not stay ther anyway!” (Doctor 4)*

*“I'm going to talk about a specific item and I do not know if it's going to be controversial, but I think the 10% [Medical Residency Bonus] is a bad thing, and it attracts a doctor who has no chance of staying inside a Health Care. I think if it was just the grant a lot of people would look for it.” (Doctor 3)*

Other factors were identified as positive for physicians’ retention to PHC from PROVAB. The work in PHC not related to stressful days of work contributed decisively to the retention of the medical professional, which denotes the quest for quality of life in these young doctors, even though they are at the beginning of their professional lives. Furthermore, the possibility of continuing to improve is understood as fundamental in a career where scientific knowledge undergoes constant changes which have a direct impact on the daily care of patients by medical professionals.

*“... it’s better than being on duty. The grant is at least very tight ...” (Doctor 1)*

*“... today it is one of the best jobs, you do not pay taxes [...] but anyway, it's a stress-free job, you get a scholarship you do not have to be on duty to run after your salary...” (Doctor 6)*

*“... not to mention the financial issue [...] for example, because I participated in the program and have the scholarship I’ve already specialized in other areas. So it allowed me to be a specialist and this I cannot forget to consider.” (Doctor 5)*

It was evident in the doctors’ speeches that, for them, choosing to work in PHC still has a negative aspect, for the fact that it’s linked with “status” and power exercised by the doctor in society since the specialties that have more emphasis on clinical care and using low density technological, are still faced, since the formation, as of lower value in relation to the role of the doctor in the community [7].

*“Now, I do not know about you, but explain to your father, your mother, who the family doctor is... My father is a doctor and my mother is a nurse. When I said that I wanted to do family medicine, it was a problem...” (Doctor 1)*

*“From all my class, I think everyone passed the residency and there are only me and another one who are in the family medicine and the others look at us saying that we are in the* PHC *because we did not pass the residency, as if we were donkeys compared to them. Family Medicine and Community is still an area that theoretically has no value for medical area, which is seen as if you are still in it, it is because you have settled down.” (Doctor 4)*

*“Like one of these days, I was scheduling exams and then they asked me what my profession was and I said I was a doctor. And then they asked me what kind of doctor I was, I thought I'm going to take the test... Family and community doctor. And the person asked: what is it?” (Doctor 1)*

### Work conditions related to the retention of the doctor in PHC

The environment, the organizational climate, and the work process directly influenced the possibilities of maintaining the professional and motivated him to propose and execute changes and qualification in his territory, even when in units with more than one team and with heterogeneous work processes.

For that matter, the combination of precarious infrastructure, untrained and disarticulated staff, in the absence of a management capable of conducting these aspects, it had the power to move away those PHC graduates, constituting a negative factor for the establishment of this professional in this level of care.

*“This “Mais Médicos” experience in the program depends a lot on where you stay and the team you stayed, right? I have nothing to complain about, but, for example, there are people I know who hated it and left the program. It is not the Basic Care, but the place where they were working. ” (Doctor 1)*

*“The team nurse, whether he is there or not, does not make a difference. So I'm a doctor, I'm a nurse, I'm everything. I'm overwhelmed. The director, despite being a good person, has no pulse to solve things, then, everyone does what they want in there...” (Doctor 6)*

Operational capacity and understanding of PHC concepts by teams and management were also determinants in the experiences developed by doctors. In these cases, PROVAB, through its professionals, worked as a positive pressure to qualify the PHC and the territory, working with the resumption of structuring concepts in relation to the performance and life of the professionals, mainly the community health agents. In the speeches, it is clear that permanent institutional education is fragile and should be implemented for all the team and not only for the medical professional.

*“I like family health, despite the insecurity. I love the work, the relationship with everyone, it's very good. But, in order to be pleasing, you need to have a job that impacts the population. You must have the whole team training, from the health agent to the director of the health center...”. (Doctor 2)*

*“One of the unscheduled functions of PROVAB and Doctors is that you have to explain what Primary Care is to everyone on the team.” (Doctor 3)*

One of the focuses of reflection in the group was the qualification of access for the populations linked to the teams of the doctors. In this perspective, the implantation of the reception demanded urgent intervention, which was determinant for the professional to exercise his capacity of leadership in the team, in search of qualification of the health care. It is noteworthy that most of the health units of doctors, at the time of the focal group, were adapting to a process of welcoming the patient, according to the patients ‘needs.

*“I think today I'm in the middle between the old model and the ideal of working and I'm managing to schedule the reception issue because I work in a Mixed Unit [FHS and Maternity] and it attracts a lot of people. The reception takes that employee who did not work and makes him work; so there is so much resistance.” (Doctor 1)*

*“I have nothing to complain about... how it works... PMAQ [Service Quality Improvement Program] came, that's when I started to organize the service and actually access it. First, with the suppressed demand you cannot do anything: 30 patients a day and look there, outside the prescriptions. So, you worked like that, looking like a pack-donkey... Over time, this was changing, and I started doing the programs correctly. It started to change things and I understood what PSF really was.” (Doctor 1)*

The value and the receipt of the scholarship on time and the need for professional stability both for doctors and the whole PHC team, as a strategy to strengthen the SUS were essential factors for the doctor’s stay in PHC, evidenced in the following statements:

*“The word* retention *is very much related to the enhancement and strengthening of* PHC*. For the professional wanting to settle it is necessary to have an ongoing career plan, with encouragement and the whole team feeling valued. That's the most important thing to stay.” (Doctor 2)*

*“... and if I also had career stability I would go on as a family doctor.” (Doctor 4)*

*“...we have a salary which is compatible with our work. The people who are linked to the city hall do not have this and it works as a cooperative. The other doctor who was there, she had to be on duty, so she could get the same thing. I know that if this scholarship was a steady job of the PSF, no doctor would leave...” (Doctor 6)*

In the doctors´ view, the lack of assurance of continuity of the professionals in the PMM established by the characteristics of the current policy may lead to a deconstruction of the qualification that was sought in recent years, with serious damage to the population and health indicators. There is a widespread fear that doctors who are now doing work compatible with the principles of PHC and FHS will be replaced by doctors hired to assist free demand, which will hamper the linkage, longitudinality and completeness of health care in those territories, achieved after the retention of physicians in the teams. These aspects are evidenced in the following statements:

*“...I'm scared to death because we could be responsible for the change.” (Doctor 1)*

*“I'm afraid of who is about to come. I have a colleague who passed the residency and left and so far there is nobody to fill the vacancy.”(Doctor 3)*

*“I'm watching the movie of Basic Care from the beginning to the end. With the arrival of the cooperative doctors who, sincerely, assist 26 patients per shift...” (Doctor 4)*

### The bond as a singular component of the retention

The possibility of making a difference during the disease course of the patient and their family, educating them to have more autonomy in the care of their own health and the emphasis on health promotion were also mentioned as factors of great satisfaction for the doctor and contributed for their retention. The doctors noticed a decisive change in the conduct of their care: to look after the health of the subject and not simply a sick part of the body, often with the small possibilities of regression or cure.

*“In the Unit that I work, I like the professionals and the population. And, as Doctor 3 said, on the preventive side. You see the result. We prevent diseases from appearing. I like it.” (Doctor 4)*

*“I like to work, I will not lie. I went into college longing to do psychiatry. I thought it was so beautiful and then I came out loving surgery. Here is so much cooler and also less tiring. I think that doing surgery is much worse than what I do here. You have a job that doesn’t have a routine and then you do not hear the same things because it is an ambulatory, and then you are in the child's health, another time you are in another area, so if you do not do the same thing every day, this is also very cool.” (Doctor 1) “My intention was to be a vascular surgeon. And I realized that my pleasure to see an amputated leg became avoiding the amputated leg. Today, I have more pleasure in avoiding that person to have a diabetic foot, I do not know, and when I go to someone's house then I say, it's my mission…” (Doctor 1)*

The establishment of the bond between the team and the community, expressed in the pleasure found in the home visits and in the approach beyond medicine with the patients and their families contributed greatly to this change of attitude of caring for the professional and the team linked to it.

*“What is most satisfying within the PSF is the bond with the community. Guys, I used to hate visiting. Today I go, I sit in the house of the little lady, There, there's bread, coffee and other things. Then they say, “Doctor, can you see whatever my examination?”... Then I say, “Bring it so that I can see it.” The home visit, this bond that you have with the community, is very cool!” (Doctor 1)*

*“I've always wanted to do gynecology and obstetrics, and one day I'll do it. But every year, I'm postponing it mainly because of the linking with the community. It is very gratifying to see that the patient you treated has worked and today is fine and you can change the thinking of the population... It is worth it!” (Doctor 5)*

Thus, the opportunity provided by PROVAB for the growth of these doctors in the technical and subjective fields of their life is evident. In these cases, the experience in Primary Health Care has a preponderant role in the training of a professional more compatible with the needs of the population.

*“I think it's really professional growth...” (Doctor 6)*

*“... almost everyone graduated and went straight to PROVAB. So, it was the prospect of professional growth. My base as primary care physician was in PROVAB.” (Doctor 3)*

Therefore, the results show that, in the experience of this group of doctors who took part in the research, there were several factors that, together, contributed to the retention of a doctor in the PHC of Brazil, as they were systematized in the figure below:

**Figure:**
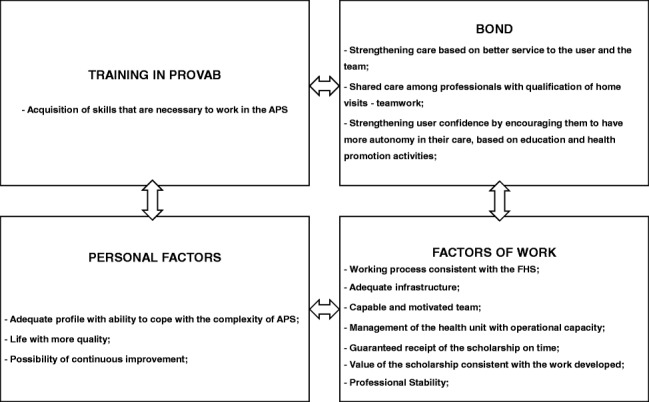
Doctors´ retention factors in PHC in the PROVAB experience

## Discussion

In accordance with the professionals who participated in this study, the PROVAB was the first great experience in PHC in their lives, as also illustrated in a study by Lima et al. with other PROVAB doctors [23]. It is clear from the findings that PHC can be a working locus desired by medical professionals when they find meaning in their actions and possibilities of transformation and improvement of care based on the close relationship and bond with users and teams. The emphasis on education and health promotion were mentioned as factors of great satisfaction among physicians, with the best case scenario of their perception that they are making a decisive change in the way health care provision for subjects and not for a sick part of the body, allows greater coherence with their true mission.

We also observed how powerful the approach of providing care with uniqueness in the extramural situations is as well as in taking into account the daily life of people, when compared with individual and outpatient appointments [24]. This expansion of care beyond Health Units, which is part of the FHS, promotes an effective care guided by logic in view of the social determinants that permeate people’s lives, as well as contributing to the establishment and strengthening of link between team and patients.

Accordingly, it was through the use of lightweight technology [25, 26] that the encounter between professionals and patients took place, where the bonds were established and strengthened and where the expanded view to the user’s life context could be included in the construction of therapeutic projects that are more in agreement with life, thus predisposing to increased integrality of care. This way of caring is consistent with the authors who say that taking care of others involves the understanding of human suffering, of getting sick beyond the biological aspect, and it is where living work becomes effective, the one that creates possibilities based on the singularity, using the tools available at the work of the physicians [7, 25, 26].

Thus, it was the living work that began to dictate the soul of the health services where teams of these doctors are allocated and where the technical-scientific knowledge, in a structured way, was made available as one of the alternatives, taking into consideration the will, the understanding and the living of those who are in need of care and attention [27, 28].

Based on the expanded view on the taxonomy of health needs [26, 27], it was observed that the work with bond and commitment enhances the act of caring, creating a connection between individual needs and social dimensions of the patients, stimulating teams and professionals to change practices, starting with the act of embracing the users and their families.

The improved access provided by the interaction of physicians with the users based on their technological toolbox (hard, lightweight-hard and lightweight technologies) [26] determined and modulated how users received assistance to their health needs since the moment they entered the Health Units. Expanded access movements brought users into the “their stories”, preventing them from being only spectators of their care. This was determinant for physicians to effectively provide extended care, seeking results based on the emphasis on promoting a healthier life and preventing primary illness or even secondary diseases to those that already have a prior one. Thus, despite all the challenges to be faced, doctors and their teams have found feasible ways to implement care management tools such as advanced access, which allows a closer approximation to the precepts of primary health care [29].

The fact that physicians did not feel prepared to work in PHC soon after graduating, because they understood that in the undergraduate period the experiences to develop competences to act in this level of attention were insufficient, is in line with works that show that the great majority of medical students are trained since the moment they enter the undergraduate program only in their own specialized environment, with the hospital being the most frequent locus of this training [30–32]. This challenge doesn’t only belong to Brazil as a nation which proposes a Unique Health System demanded by PHC. Mash and von Pressentin [33] when studying family and community medicine in this country reached some conclusions that are similar to Brazil, as common characteristics to qualification and retention of general physicians in PHC.Public service challenges stand out in order to balance the necessities of Family medicine professional training, aiming to qualify and suit them according to the population’s health needs, as well as becoming competent in a group demanding multiple tasks and competences that go beyond clinical knowledge and the provision of services to the whole population who depends on public health assistance.

Thus, for this group of doctors from PROVAB program, it is understood that training for PHC in undergraduate programs, in the logic of interaction between teaching, service and community is still something to be pursued by some medical schools, aiming at the training of professionals who are more aligned with the health needs of the population [27, 29, 34]. From this perspective, the PROVAB had the power to create skills that were not very well addressed at the undergraduate level, such as those related to teamwork and access quality management, which were determinant for the professional’s work and the decision to stay in PHC. In 2010 WHO developed a wide range of guidance which were based on the Best available evidence. PROVAB and PMM both have a strong influence on two of these categories: education/financial incentives and personal/professional support, since the fresh graduated doctors have frequent supervision from a medicine doctor with long experience, they also have the commitment to stay in post-graduation formative activities, for the fact that the program provides fair and safe wage [1].

In fact, the complexity of PHC users and communities found by the physicians [34] represented challenges that required the professionals to seek alternative paths with their team and in the management of the territory. Physicians face in their daily life the need to identify the role they are playing, which require them to be proactive, resilient and courageous to face complex problems every day [34]. In this way, it was identified in this study that, besides remuneration and motivation, it is essential for physicians to have a profile that is adequate for the complexity of PHC. This profile, which was not developed during undergraduate training, was provided to the program’s physicians through real and transformative experiences with the teams and their territories, stimulated by the training component of PROVAB.

With these results we conclude that the practice scenarios related to PHC are necessary for the development of competences related to general practitioners, since it is shown at this level of care that about 80% of the population’s health needs must be accomplished [35] and it is where a large part of the graduates will work, even temporarily, before entering the medical residency. Reid et al. [36] observed that the participants in the Community services program in South Africa were also submitted to a Professional improvement during the 12 months of practicing, in these cases there was a probabibilty of retention of these professionals in deprived communities which was twice bigger, and one of the main objectives of this program in South Africa, which is to guarantee a better provision of health services to the citizens, was achieved.

We observed that the PROVAB played an important role in showing the reality of work to physicians who are inserted in family health teams, as a real and compensatory possibility, allowing physicians to walk their path not only by market influences but taking into consideration their vocation and making them reflect beyond the factors of future remuneration and recognition before their peers and society in general [37, 38], leaving them better prepared to deal with the real needs of the population.

In addition to the still pressing challenges in medical training, this study demonstrated that the conditions and work environment related to organizational climate consistent with the FHS work process and the possibility of permanent improvement with the perspective of future stability were determinant for the professionals. By postponing entry into the specializations offered by medical residencies, changing the course of their lives, they understand that it is in PHC that they could get settled as long as they have adequate conditions for the development of their activities. The assurance of receiving the salary was also a strong motivation for the permanence of these professionals in PHC, giving them peace and stability to face the complexity of the daily life in the FHS. We observed in the speeches that, with the lack of adequate conditions for the development of PHC, the subsidy became the main reason for staying in the program. In these situations, they tend to perpetuate the factors that led the professionals to enter the Program, discouraging them to remain in PHC, a situation also observed in another study [23, 39]. A limitation of the study was the lack of participation of all the doctors that had precedence over PROVAB to PMM, which might had affected the inclusion of other positive perceptions related to the possible advances in the formative perspective of the Program, as well as the retention of the professionals in this field, once the study suggested analyzing the factors and motivations that led doctors to take this decision. As for the limited number of participants, Mash and Reid [20] defend the focal group method, asserting that it is particularly useful approach to explore the knowledge and the experiences of the people, making it possible to be used not only what people think, but how they think and why they think, achieving a deep comprehension about the studied object. About the size of the sample they affirm that the chosen groups to take part in the focal group don’t need to be so big as in quantitative studies and that a sample in size that goes from five to 15 interviewed is adequate for obtaining a good range of answers and experiences, so that a level of saturation can be reached; besides that no production which is considerably new may be produced. In this study, all parameters were sought and achieved successfully.

Natal was chosen as the scenario of the research for the fact that it has been the city where the doctors have remained in the PHC within the state of Rio Grande do Norte. This happens because it is the capital city, and it provides a better quality of life for the doctors and their families as well as continuity with regard to the studies. The situation is similar in other large cities. Therefore, even considering all these positive factors for the retention of the doctors at the PHC in a capital city, the locations of the health units are similar to rural areas of the country, with a great difficulty in solving the problems of the population, due to the distance of these places to major hospitals and the level of education of the residents. These factors are crucial to the low numbers of doctors in relation to their basic necessities, especially in extremely poor regions.

The findings allow inducement of the factors that are essential for the doctors´ retention in Brazil’s PHC, guiding and supporting solutions to this problem, still so far from being resolved by the current public policies, and it is necessary to continue with other studies in the other regions of the country to complement and reinforce the results of this research.

As a recommendation of the research and considering the moment of transition in the Brazilian medical training, as a consequence of the law of the PMM, it is necessary that there is a prospective follow-up of the insertion of the recently graduated Brazilian professionals in this program, since, PROVAB was completed in the second half of 2017. [40–43].

## Conclusions

We found that the newly trained physicians started to work in PHC with a lack of skills, mostly due to their previous training in HEI, whose curriculum focused on privileging the specialties and the hospital locus, differently from the directions of the National Curricular Guidelines. In this regard, the results showed how important the year of immersion as a PHC doctor was, with maintenance of the formative logic.

PROVAB acted as the main training in PHC, acting as a complement for those who had not had this experience in undergraduate schools. It also showed the importance of teaching, service and community interaction in order to promote autonomy among professionals and at the same time to keep them supported at a distance, either through supervision or by specialization in Family Health. The study identified that interdisciplinarity and teamwork, when exercised to the fullest extent, give professionals real meaning to their mission in the practice of medicine, sharing responsibilities, and leaving this challenge lighter and more pleasurable. Moreover, it increases the resolution of population problems and contributes to PHC, being able to show its potential and richness in face of the users’ health needs.

As a very evident result, there is instability linked with the permanence of these professionals in the territory that, according to their perception, will generate a discontinuity of the work they developed with their teams and, consequently, great harm to all the population involved with them.

It was clear that doctors who decide to live their life in the PHC have important challenges to overcome. The understanding of the relevance of these professionals, qualified and committed to the population, needs to be strongly debated in all spheres. The living work of PHC is not carried out, however, based on just one view; the investment is fundamental and it has to be extended to all professional areas, considering the immense inequities present in Brazil.

Finally, despite all the challenges faced by the PROVAB throughout its course, the program has had a unique contribution to the qualification of PHC, as well as to the birth of a new logic of medical training in Brazil. Its effective contribution to the consolidation of the PMM, which is still under construction, is a major movement of resistance to the disarticulation and deconstruction of the SUS observed every day in Brazil.

## Abbreviations

FHS: Family Health Strategy
HEI: Higher Education Institutions
PHC: Primary Health Care
PMAQ: National Program for Improving Access and Quality of Primary Care
PMM: Mais Médicos (More Doctors) Program
PROVAB: Program to Value Primary Healthcare Professionals
SUS: Unified Health System (Sistema Único de Saúde)
